# *cat*RAPID *signature*: identification of ribonucleoproteins and RNA-binding regions

**DOI:** 10.1093/bioinformatics/btv629

**Published:** 2015-10-31

**Authors:** Carmen Maria Livi, Petr Klus, Riccardo Delli Ponti, Gian Gaetano Tartaglia

**Affiliations:** ^1^ Centre for Genomic Regulation (CRG), The Barcelona Institute of Science and Technology, Dr. Aiguader 88, Barcelona 08003, Spain,; ^2^ Universitat Pompeu Fabra (UPF), 08003 Barcelona, Spain and; ^3^ Institució Catalana de Recerca i Estudis Avançats (ICREA), 08010 Barcelona, Spain

## Abstract

**Motivation:** Recent technological advances revealed that an unexpected large number of proteins interact with transcripts even if the RNA-binding domains are not annotated. We introduce *cat*RAPID *signature* to identify ribonucleoproteins based on physico-chemical features instead of sequence similarity searches. The algorithm, trained on human proteins and tested on model organisms, calculates the overall RNA-binding propensity followed by the prediction of RNA-binding regions. *catRAPID signature* outperforms other algorithms in the identification of RNA-binding proteins and detection of non-classical RNA-binding regions. Results are visualized on a webpage and can be downloaded or forwarded to *cat*RAPID *omics* for predictions of RNA targets.

**Availability and implementation:** *cat*RAPID *signature* can be accessed at http://s.tartaglialab.com/new_submission/signature.

**Contact:** gian.tartaglia@crg.es or gian@tartaglialab.com

**Supplementary information:** Supplementary data are available at *Bioinformatics* online.

## 1 Introduction

RNA-binding proteins (RBPs) use RNA-binding domains (RDs) to recognize target RNAs and to regulate co-/post-transcriptional processes. Examples of classical RDs include RNA-recognition motif (RRM), double-stranded RNA-binding domain (dsRRM), K-homology (KH), RGG box and the Pumilio/FBF (PUM) domain (Lunde *et al.*, 2007). In addition to classical RDs, recent experimental studies on HeLa (Castello *et al.*, 2012), HEK298 (Baltz *et al.*, 2012) and mESC (Kwon *et al.*, 2013) cells, indicate that a number of RNA-interacting proteins contain non-classical RDs (ncRDs) for which annotation is not yet available. Discovery of new RDs is a challenging task: domain-detection tools, such as HMMER (Finn *et al.*, 2011) and BLAST (Camacho *et al.*, 2009) rely on sequence similarity searches to identify annotated RDs and fail to recognize newly discovered RBPs. Similarly, other methods such as RNApred (Kumar *et al.*, 2011) predict RNA-binding ability using features of annotated RDs that might be different in ncRDs. Alternatives to identify RNA-binding regions include BindN+ (Wang *et al.*, 2010), PPRInt (Kumar *et al.*, 2008) and RNAbindR+ (Walia *et al.*, 2014), but the algorithms have been trained to identify single amino acids and not contiguous regions. *cat*RAPID *signature* overcomes these limitations by (i) predicting the propensity of a protein to interact with RNA and (ii) identifying RNA-binding regions through physico-chemical properties instead of sequence patterns. The algorithm is an extension of the *cat*RAPID approach (Bellucci *et al.*, 2011) to predict protein-RNA interactions and the *clever*Suite algorithm (Klus *et al.*, 2014) to classify protein groups using physico-chemical features.

## 2 Algorithm and performances

To build *cat*RAPID *signature* we exploited a number of physico-chemical properties reported in our previous publication (Klus *et al.*, 2014):

- We used each physico-chemical property [e.g. structural disorder (Castello *et al.*, 2012)] to build a *signature*, or profile, containing position-specific information arranged in a sequential order from the N- to the C-terminus;
- We computed Pearson correlation coefficient between signatures of annotated human RDs and same-length regions taken from RNA-binding proteins as well as negative controls (Supplementary Table S1 and online Documentation);
- We identified a number of discriminating physico-chemical properties, their associated RDs and correlation cutoffs (Supplementary Table S2 and online Documentation).

For each protein, we calculated the fraction of residues with correlation coefficients above the cutoffs that are associated with physico-chemical properties and RDs (Table S2; online Documentation), which we then used to train *cat*RAPID *signature.* Using a Support Vector Machine with RBF-kernel (online Documentation), we built a method for the (i) identification of ribonucleoproteins and (ii) prediction of RNA-binding regions:

- *cat*RAPID *signature* shows an AUC = 0.76 for discrimination of 950 RBPs from 950 negative cases (10-fold cross-validation; Supplementary Fig. S1, Supplementary Data). On an independent test set (Table S3) comprising 47 mouse proteins harboring ncRDs and same number of negatives (Kwon *et al.*, 2013), we obtained accuracy = 0.71, sensitivity = 0.70, specificity = 0.72 and precision = 0.70. By contrast, conventional pattern recognition methods such as HMMER and BLAST show poor sensitivity (Table S3). Our algorithm outperforms RNApred in both specificity and precision (0.25 and 0.52, respectively; Table S3). Moreover, *cat*RAPID *signature* reliably detects ribonucleoproteins across different kingdoms, including *M. pulmonis, E. coli, C. albicans, S. cerevisiae, A. thaliana and A. oryza* (Supplementary Fig. S2; online Documentation).
- The training for the identification of RNA-binding regions has been done on 1115 annotated RNA-binding regions. As negative counterpart we randomly selected 1115 non-binding regions of the same length from each RBP (AUC = 0.80 in 10-fold cross-validation; Supplementary Fig. S1). On 102 ncRDs versus 102 negative mouse proteins, *cat*RAPID *signature* outperforms other algorithms: accuracy = 0.67, sensitivity = 0.76, specificity = 0.60 and precision = 0.65 (Supplementary Table S4). By contrast, *RNABindR + *shows accuracy = 0.48, sensitivity = 0.53, specificity = 0.42 and precision = 0.48. Similar performances were obtained for BindN + and PPRInt (Supplementary Table S4). In addition, we observed high performances on a protein dataset whose RNA-binding sites have been determined through X-ray and NMR (Supplementary Fig. S3 and online Documentation).

## 3 Server description and example

**Fig. 1.**
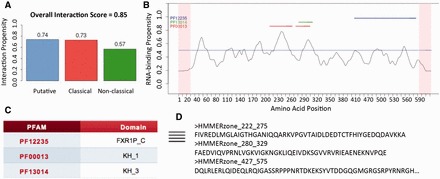
*RNA-binding ability of Fragile X Mental Retardation Protein FMRP.* (**A**) The server reports the propensity of FMRP for the putative (0.74), classical (0.73) and non-classical (0.57) RBP classes, as well as an overall prediction score (0.85); (**B**) The profile shows protein regions and their propensity to interact with RNA. *catRAPID signature* correctly identifies two peaks corresponding to the central KH domains, a region in the RGG box [amino acids 527-552] at the C-terminus (Ascano *et al.*, 2012) and a recently discovered RD at the N-terminus (Myrick *et al.*, 2015)**.** (**C**) Annotated RDs are shown in a table and linked to PFAM webpages; (**D**) Annotated and predicted RNA-binding sequences can be downloaded and/or forwarded to *cat*RAPID *omics* (Agostini *et al.*, 2013) for further analysis

The input of the server is a FASTA sequence. To illustrate the output with an example, we studied the RNA-binding ability of Fragile X Mental Retardation Protein FMRP. *cat*RAPID *signature* predicts that FMRP binds to RNA (overall interaction score = 0.85; Fig. 1A; Fig. S4) and correctly identifies two peaks corresponding to the KH domains and one peak in the RGG box (Ascano *et al.*, 2012) [Fig. 1A,B and C; ‘classical’ score = 0.73]. In addition, *cat*RAPID *signature* indicates that the N-terminus (amino acids 1-215; Fig. 1B) has RNA-binding ability (‘putative’ score = 0.74), which is in agreement with very recent evidence revealing the presence of a novel KH domain (Myrick *et al.*, 2015). Comparing experimental targets [number of PAR-CLIP binding sites ≥ 1] (Ascano *et al.*, 2012) with transcriptome-wide predictions of FMRP N-terminus [amino acids 1–215; Fig. 1D] (Agostini *et al.*, 2013) we observed a significant enrichment in predicted interaction propensities (*P*-value < 1 ^−^^9^ calculated with Kolmogorov–Smirnov test on 105 × 10^3^ transcripts of which 7 × 10^3^ positives), which suggests that the N-terminus contributes to the RNA-binding ability of the full-length FMRP.

## 4 Conclusions

As newly discovered RDs are not annotated, traditional domain-detection tools fail their identification. *cat*RAPID *signature* addresses this limitation by detecting binding regions through physico-chemical features. Our algorithm will be helpful to investigate components of ribonucleoprotein complexes and to identify RNA-binding regions.

## Supplementary Material

Supplementary Data
