# Supplementary material for: catRAPID signature: identification of ribonucleoproteins and RNA-binding regions
Source: Bioinformatics. 2015 Oct 31;32(5):773–5. doi: 10.1093/bioinformatics/btv629 (PMC4795616; doi:10.1093/bioinformatics/btv629)
Supplement: Supplementary Data [file btv629_supplementary_data.zip › btv629-Livi_supplementaryReviewded.pdf]

Supplementary Information

Application Note

*cat*RAPID *signature*: Identification of Ribonucleoproteins and RNA-Binding Regions

Carmen Maria Livi[1,2], Petr Klus[1,2], Riccardo Delli Ponti[1,2] and Gian Gaetano Tartaglia[1,2,3*]

[1] Centre for Genomic Regulation (CRG), The Barcelona Institute of Science and Technology, Dr. Aiguader 88, Barcelona 08003, Spain.

[2] Universitat Pompeu Fabra (UPF), 08003 Barcelona, Spain.

[3] Institució Catalana de Recerca i Estudis Avançats (ICREA), 23 Passeig Lluís Companys, 08010 Barcelona, Spain

* Corresponding author: Gian Gaetano Tartaglia. Telephone +34 933160116. Email: gian.tartaglia@crg.es.

This document includes the following information: Tables S1, S2, S3 and S4; Figures S1, S2, S3 and S4;

Supplementary Tables

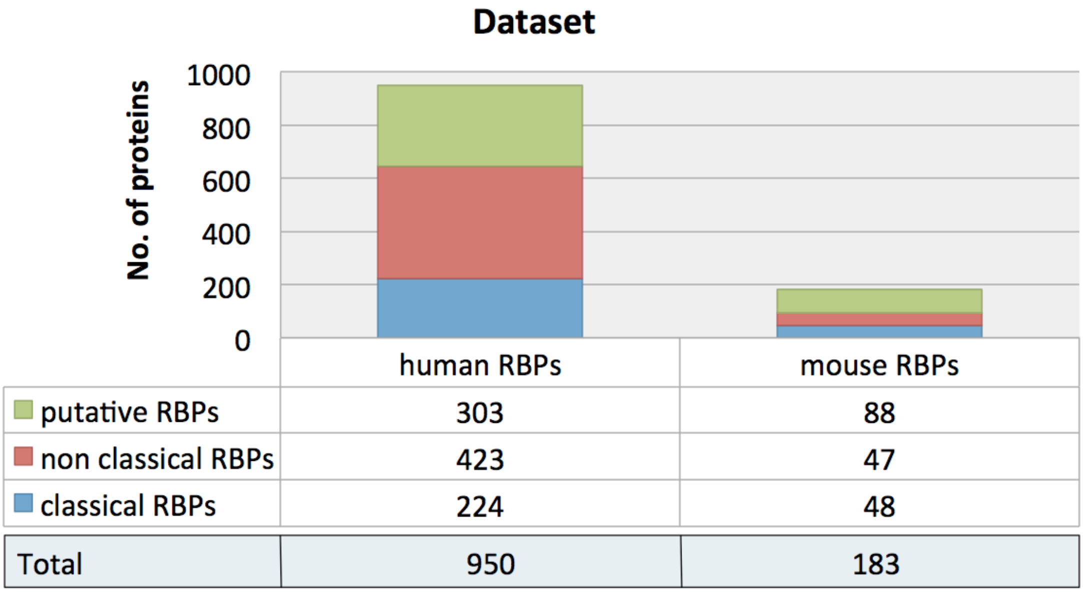

	human RBPs	mouse RBPs
putative RBPs	303	88
non classical RBPs	423	47
classical RBPs	224	48
Total	950	183

Table S1 RNA-binding proteins (RBPs) were taken from studies on human HeLa (Castello et al.; 2012) and HEK293 (Baltz et al., 2012), and from mouse embryonic stem cells mESC (Kwon et al., 2013). CD-HIT (Li et al., 2006) was employed to reduce sequence similarity to 30% on the joined datasets (HeLa, HEK293 and mESC). As non-RNA-binding proteins we define the fraction of non RNA-bound proteins from HeLa (Castello *et al.*, 2012) [total of 2813 proteins; <30% sequence similarity]. Classical RBPs are defined as proteins possessing classic RNA-binding domains (RDs), non-classical RBPs are proteins possessing non-classical RNA-binding domains (ncRDs) and putative RBPs are the fraction of proteins without annotated RNA-binding domains. Further details about the classification can be found in (Castello et al.; 2012).

Physico-chemical property	Reference domains from PFAM
Nucleic Acid Binding-interface_close-1 (Terribilini *et al.*, 2006)	SEC13_HUMAN/202-244,RBBP5_HUMAN/15-52,<br>E9PB61_HUMAN/115-184, APAF_HUMAN/647-685
Nucleic Acid Binding-HB (Lewis *et al.*, 2011)<br>Nucleic Acid Binding-interface (Lewis *et al.*, 2011)<br>Nucleic Acid Binding-interface_close+1 (Terribilini *et al.*, 2006)	SEC13_HUMAN/3-41,PELO_HUMAN/271-370,<br>GAR1_HUMAN/46-203,SRSF6_HUMAN/112-206<br>SF3A2_HUMAN/41-91,CELF1_HUMAN/403-473,<br>A0A024R4E5_HUMAN/586-637
Aggregation (Pawar *et al.*, 2005)	H0YAS6_HUMAN/76-147, CELF1_HUMAN/124-186,<br>SURF6_HUMAN/135-351,U3IP2_HUMAN/231-269,
Aggregation (Conchillo-Solé *et al.*, 2007)<br>Aggregation med (Fernandez-Escamilla *et al.*, 2004)<br>Aggregation high (Fernandez-Escamilla *et al.*, 2004)	FAKD1_HUMAN/779-838, NOP58_HUMAN/253-401,<br>WDHD1_HUMAN/3-41, APAF_HUMAN/1075-1113,<br>RBM4_HUMAN/160-177,ZCH14_HUMAN/906-923,<br>RBM4_HUMAN/160-177,ENSP00000361327_13-214,<br>MAZ_HUMAN/438-488, LN28B_HUMAN/127-144
Hydrophobicity (Sweet and Eisenberg, 1983)<br>Hydrophobicity (Bull and Breese, 1974)<br>Hydrophobicity (Abraham and Leo, 1987)<br>Hydrophobicity (Black and Mould, 1991)<br>Hydrophobicity (Fauchere and Pliska, 1983)<br>Membrane-buried preference (Argos *et al.*, 1982)<br>Transmembrane regions (Nakashima *et al.*, 1990)	XRCC6_HUMAN/558-607, WDR1_HUMAN/224-262<br>PLRG1_HUMAN/403-440, ZCH14_HUMAN/906-923<br>ENSP00000361327_13-214<br>NOP58_HUMAN/253-401,<br>NOP58_HUMAN/253-401, XRCC6_HUMAN/558-607<br>PI3R4_HUMAN/983-1021, HTSF1_HUMAN/291-347<br>TBL3_HUMAN/282-320
Normalized frequency of alpha-helix (Levitt, 1978)<br>Normalized frequency of alpha-helix (Burgess *et al.*, 1974)<br>Normalized frequency of middle helix (Crawford *et al.*, 1973)<br>Average relative probability of helix (Kanehisa and Tsong, 1980)<br>Normalized frequency of alpha-helix (Palau *et al.*, 1982)<br>Normalized frequency of beta-sheet (Chou and Fasman, 1978)<br>Normalized frequency of beta-sheet (Crawford *et al.*, 1973)<br>Average probability of inner beta-sheet (Kanehisa and Tsong, 1980)<br>Normalized frequency of beta-sheet (Levitt, 1978)	GAR1_HUMAN/46-203<br>SRSF6_HUMAN/112-206<br>PRP8_HUMAN/1208-1343<br>PELO_HUMAN/271-370<br>DCAF8_HUMAN/226-266<br>KTNB1_HUMAN/221-258<br>RL5_HUMAN/26-173, CSTF1_HUMAN/387-425<br>FUS_HUMAN/287-365<br>RB15B_HUMAN/420-482, TAF5L_HUMAN/259-296
Normalized frequency of coil (Nagano, 1973)<br>TOP-IDB (Campen *et al.*, 2008)<br>Average flexibility indices (Bhaskaran and Ponnuswamy, 1984)<br>UnfoldOverFold (Campen *et al.*, 2008)<br>Coil (Deléage and Roux, 1987)<br>Proportion of residues 95% buried (Chothia, 1976)<br>Percentage of buried residues (Janin and Wodak, 1978)<br>Energy transfer from out to in (95% buried) (Radzicka *et al.*, 1988)	NCBP2_HUMAN/56-116<br>SUV3_HUMAN/624-674, MINT_HUMAN/519-583<br>FAN_HUMAN/704-740<br>LN28B_HUMAN/149-166<br>GAR1_HUMAN/46-203<br>XRCC6_HUMAN/558-607, APAF_HUMAN/1034-1071<br>RBM4_HUMAN/160-177, RBM4_HUMAN/160-177<br>TBL3_HUMAN/567-591, ZFR_HUMAN/369-419

Table S2. *Physico-chemical properties and RNA-binding domains used to discriminate RBPs and RNA-binding regions.* The domain sequences have been taken from PFAM database and filtered by sequence similarity. To explain annotations with an example, CELF1_HUMAN/403-473 indicates the RRM domain located at amino acids 403-473 of protein CELF1, which is associated with 'nucleic acid binding' property taken from a previous publication (Terribilini *et al.*, 2006).

In addition to RNA-binding propensities, we found that structural disorder, aggregation and membrane domains are key-characteristics to determine protein ability to interact with transcripts, followed by secondary structure propensities:

- Structural disorder (Castello *et al.*, 2012) is intrinsically associated with low burial and large radius of gyration (Marsh, 2013);
- Strong hydrophobicity was found in the RDs of RBPs that are intrinsically prone to aggregate: a study investigating truncated constructs of TDP43 in yeast, reports that presence of the RRM2 is necessary for amyloid fibrils formation (Johnson *et al.*, 2008);
- A number of membrane proteins associate with RNA to control viral replication (Hyodo *et al.*, 2014; Sha and Luo, 1997)
- Secondary structure is a key signature of known RDs and defines the affinity for RNA-binding (Lunde *et al.*, 2007; Maris *et al.*, 2005);

Dataset	Method	AUC	Accuracy	Sensitivity	Specificity	Precision
**Human (423 non classical)**	catRAPID	0.72	0.67	0.68	0.68	0.63
	HMMER	NA	0.58	0.12	0.98	0.92
	BLAST	NA	0.50	0.10	0.62	0.20
	RNApred	0.66	0.56	0.89	0.24	0.54
**Mouse (47 non classical)**	catRAPID	0.76	0.71	0.70	0.72	0.70
	HMMER	NA	0.76	0.54	0.98	0.96
	BLAST	NA	0.39	0.14	0.63	0.26
	RNApred	0.72	0.54	0.85	0.25	0.52
**Human (303 putative)**	catRAPID	0.79	0.73	0.74	0.72	0.73
	HMMER	NA	0.56	0.10	0.98	0.87
	BLAST	NA	0.37	0.10	0.62	0.18
	RNApred	0.54	0.48	0.72	0.26	0.48
**Mouse (88 putative)**	catRAPID	0.71	0.66	0.65	0.67	0.63
	HMMER	NA	0.51	0.06	0.80	0.80
	BLAST	NA	0.32	0.02	0.63	0.05
	RNApred	0.63	0.66	0.85	0.26	0.53

Table S3. *RNA-binding ability prediction*. A machine learning approach (Pedregosa *et al.*, 2011) was employed to predict the RNA-binding propensities using correlations of discriminative RDs and associated physico-chemical properties (Table S2). Training on the human dataset (950 RBPs, 950 non-RNA-binding proteins) and performing cross-organism validation on mouse proteins, we calculated the area under the ROC curve (AUC), accuracy, sensitivity, specificity and precision of the model. Performances have been compared with: RNApred (Kumar *et al.*, 2011) a method to predict the RNA-binding ability of a protein sequences, HMMER (Finn *et al.*, 2011) a classical domain-searching tool and BLAST (Camacho *et al.*, 2009) a sequence similarity search tool. The same set of annotated domains used to train *catRAPID signature* has been used in HMMER and BLAST searches. AUCs have been calculated for *cat*RAPID *signature* and RNApred using the prediction score. As BLAST and HMMER only provide the e-value of annotated domains, the AUC could not be calculated.

Method	AUC	Accuracy	Sensitivity	Specificity	Precision
*cat*RAPID	0.84	0.67	0.76	0.60	0.65
BindN+	0.48	0.38	0.37	0.39	0.38
PPRInt	0.50	0.47	0.49	0.45	0.49
RNAbindR+	0.54	0.48	0.53	0.42	0.48

Table S4 *RNA-binding regions prediction.* A machine learning approach (Pedregosa *et al.*, 2011) was employed to identify RNA-binding regions using correlations of preselected RNA-binding domains and associated physico-chemical properties (Table S2). The training has been carried out on 1115 human annotated RNA-binding regions versus 1115 same-length regions extracted from protein regions that do not bind RNA. In the cross-organism validation we tested 102 annotated RNA-binding regions vs. 102 non-RNA-binding regions from mouse. Performances were evaluated calculating AUC, accuracy, sensitivity, specificity and precision and compared with three methods that predict RNA-binding residues in proteins: BindN+ (Wang *et al.*, 2010), PPRInt (Kumar *et al.*, 2008) and RNAbindR+ (Walia *et al.*, 2014). In the analysis of RNABindR+, BindN+ and PPRInt performances, we considered as positive the ncRD predicted to contain more binding residues than its negative counterpart (both regions have same sequence length). We would like to stress that BindN+, PPRInt and RNAbindR+ have been designed to predict the RNA-binding ability of individual residues in protein sequences while *cat*RAPID *signature* identifies RNA-binding regions, i.e. contiguous binding domains.

Supplementary Figures

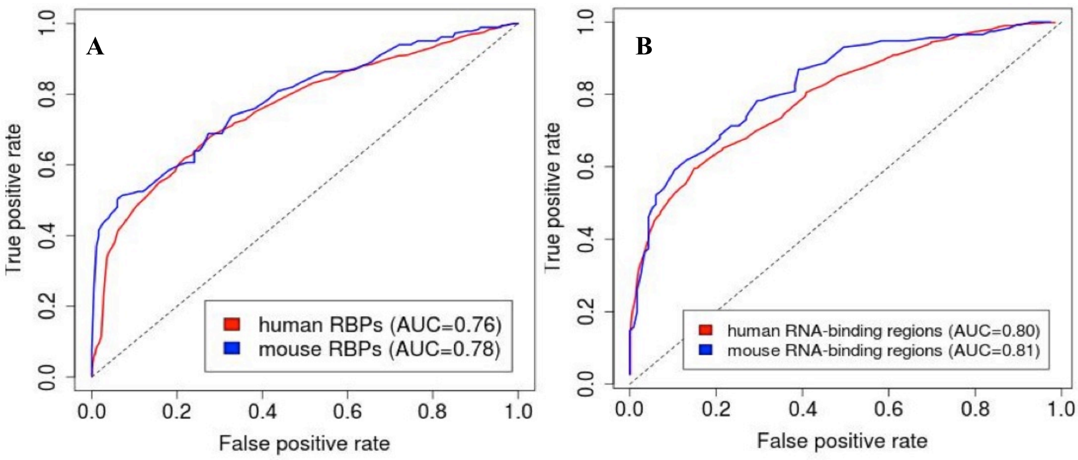

Figure S1. *catRAPID signature at a glance.* A) The plot shows the performance of the first module in predicting the RNA-binding proteome, in a 10-fold cross-validation for human (blue line) and for mouse (independent test set; red line). B) The plot shows the performance of the second module in identifying RNA-binding regions, for human (blue line) performing a 10-fold cross-validation and for mouse as independent test (red lines).

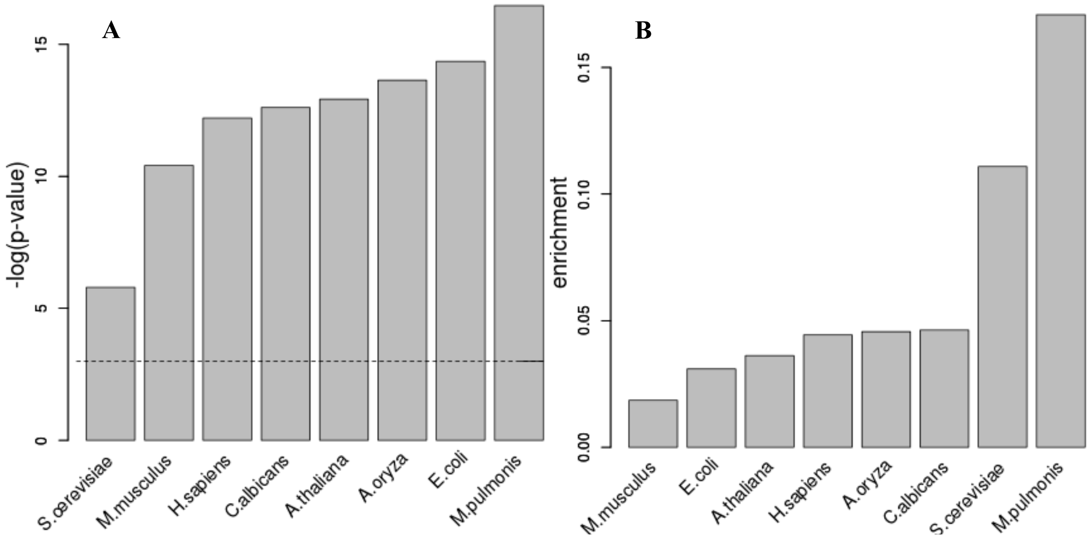

Figure S2. Performances of *catRAPID signature* on model organisms. Using *cat*RAPID *signature*, we predicted the RNA-binding propensities of the following proteomes: *Mus musculus, Homo Sapiens, Candida albicans, Saccharomyces cerevisiae, Mycoplasma pulmonis, Escherichia coli, Arabidopsis thaliana and Aspergillus oryza*. A) The plot indicates significance (-log of p-value) of the GO-term "*ribonucleoprotein complex" (GO:0030529; cellular component)*. B) The barplot shows the enrichment associated. The significance threshold of 0.01 is shown as dotted line.

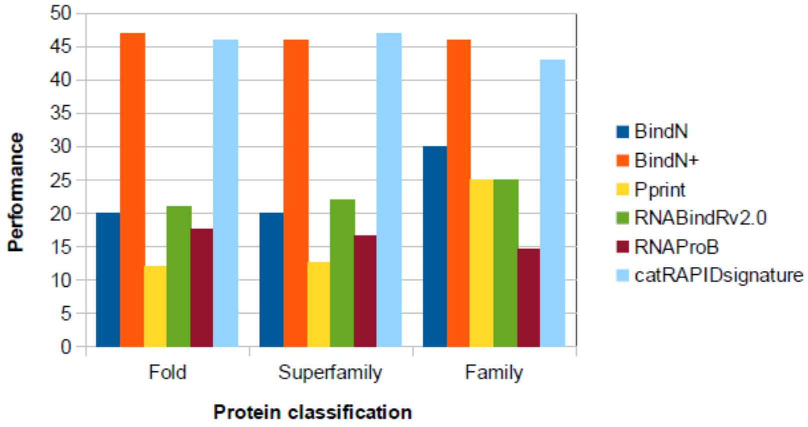

Figure S3. *catRAPID signature vs residue-binding predictors.* We calculated performances of BindN, BindN+, PPRInt. RNAbindR, RNAProB (Cheng *et al.*, 2008) and *cat*RAPID *signature* on a set of proteins whose RNA-binding sites have been validated with X-ray and NMR techniques. As in a recent work (Nagarajan and Gromiha, 2014), we used three protein classes "Fold", "Superfamily" and "Family" and the formula (sensitivity+specificity)/2 to estimate the performances.

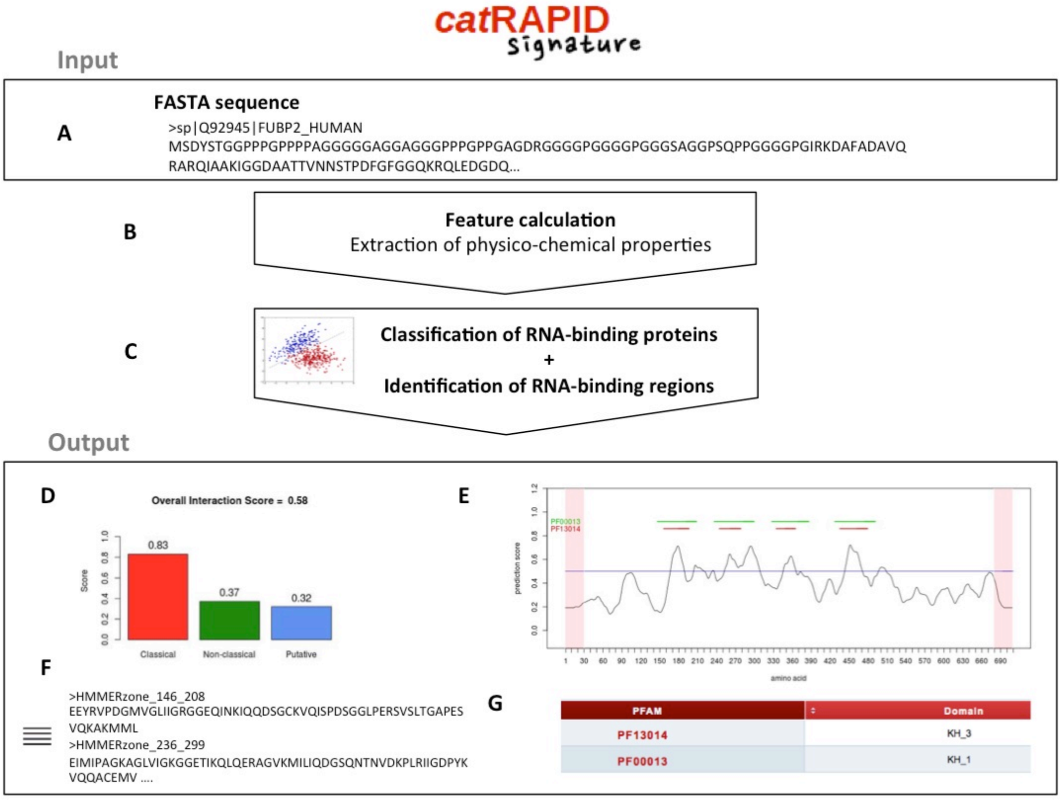

Figure S4. *catRAPID signature workflow and output.* (A) *catRAPID signature* is based on primary structure information and accepts protein sequences in FASTA format. (B) Known RNA-binding domains (RDs) and physico-chemical properties are used to classify protein sets. (C) The first module discriminates RBPs: the classifier, trained on correlations of selected RDs and associated physico-chemical properties, assigns an overall interaction score, indicating whether the protein interacts with RNA or not. Once the protein is predicted to be RNA-binding (overall interaction score ≥0.5), the second module is used to identify potential RNA-binding regions: the classifier assigns a score to each amino acid, indicating its propensity to be part of an RNA-binding region. The results of our predictions are visualized as follows: (D) The first module provides the overall interaction score along with a graph plot showing three scores (ranging from 0 to1) for classical, non-classical and putative class assignments. (E) The output of the second module is a plot visualizing protein regions that are prone to interact with RNA. The amino acid sequence is plotted along the x-axis and the prediction score along the y-axis (positive if the score is >0.5). If present, annotated RDs are shown as horizontal bars above the profile plot. (F) Predicted and detected binding regions can be downloaded and forwarded to *cat*RAPID *omics*. (G) Annotated RDs are shown in a table and redirected to PFAM webpages.

References

Abraham,D.J. and Leo,A.J. (1987) Extension of the fragment method to calculate amino acid zwitterion and side chain partition coefficients. *Proteins*, **2**, 130–152.

Argos,P. *et al.* (1982) Structural prediction of membrane-bound proteins. *Eur. J. Biochem. FEBS*, **128**, 565–575.

Bhaskaran,R. and Ponnuswamy,P.K. (1984) Dynamics of amino acid residues in globular proteins. *Int. J. Pept. Protein Res.*, **24**, 180–191.

Black,S.D. and Mould,D.R. (1991) Development of hydrophobicity parameters to analyze proteins which bear post- or cotranslational modifications. *Anal. Biochem.*, **193**, 72–82.

Bull,H.B. and Breese,K. (1974) Surface tension of amino acid solutions: a hydrophobicity scale of the amino acid residues. *Arch. Biochem. Biophys.*, **161**, 665–670.

Burgess,A. *et al.* (1974) Analysis of conformation of amino acid residues and prediction of backbone topography in proteins. 239ñ286.

Camacho,C. *et al.* (2009) BLAST+: architecture and applications. *BMC Bioinformatics*, **10**, 421.

Campen,A. *et al.* (2008) TOP-IDP-scale: a new amino acid scale measuring propensity for intrinsic disorder. *Protein Pept. Lett.*, **15**, 956–963.

Castello,A. *et al.* (2012) Insights into RNA biology from an atlas of mammalian mRNA-binding proteins. *Cell*, **149**, 1393–1406.

Cheng, C.W. *et al.* (2008) Predicting RNA-binding sites of proteins using support vector machines and evolutionary information. *BMC Bioinformatics*. **9** Suppl 12:S6.

Chothia,C. (1976) The nature of the accessible and buried surfaces in proteins. *J. Mol. Biol.*, **105**, 1–12.

Chou,P.Y. and Fasman,G.D. (1978) Prediction of the secondary structure of proteins from their amino acid sequence. *Adv. Enzymol. Relat. Areas Mol. Biol.*, **47**, 45–148.

Conchillo-SolÈ,O. *et al.* (2007) AGGRESCAN: a server for the prediction and evaluation of 'hot spots' of aggregation in polypeptides. *BMC Bioinformatics*, **8**, 65.

Crawford,J.L. *et al.* (1973) The reverse turn as a polypeptide conformation in globular proteins. *Proc. Natl. Acad. Sci. U. S. A.*, **70**, 538–542.

DelÈage,G. and Roux,B. (1987) An algorithm for protein secondary structure prediction based on class prediction. *Protein Eng.*, **1**, 289–294.

Fauchere,J. and Pliska,V. (1983) Hydrophobic parameters pi of amino-acid side chains from the partitioning of N-acetyl-amino-acid amides. **18**, 369ñ375.

Fernandez-Escamilla,A.-M. *et al.* (2004) Prediction of sequence-dependent and mutational effects on the aggregation of peptides and proteins. *Nat. Biotechnol.*, **22**, 1302–1306.

Finn,R.D. *et al.* (2011) HMMER web server: interactive sequence similarity searching. *Nucleic Acids Res.*, **39**, W29–37.

Hyodo,K. *et al.* (2014) Host and viral RNA-binding proteins involved in membrane targeting, replication and intercellular movement of plant RNA virus genomes. *Front. Plant Sci.*, **5**.

Janin,J. and Wodak,S. (1978) Conformation of amino acid side-chains in proteins. *J. Mol. Biol.*, **125**, 357–386.

Johnson,B.S. *et al.* (2008) A yeast TDP-43 proteinopathy model: Exploring the molecular determinants of TDP-43 aggregation and cellular toxicity. *Proc. Natl. Acad. Sci.*, **105**, 6439–6444.

Kanehisa,M.I. and Tsong,T.Y. (1980) Local hydrophobicity stabilizes secondary structures in proteins. *Biopolymers*, **19**, 1617–1628.

Kumar,M. *et al.* (2008) Prediction of RNA binding sites in a protein using SVM and PSSM profile. *Proteins*, **71**, 189–194.

Kumar,M. *et al.* (2011) SVM based prediction of RNA-binding proteins using binding residues and evolutionary information. *J. Mol. Recognit. JMR*, **24**, 303–313.

Levitt,M. (1978) Conformational preferences of amino acids in globular proteins. *Biochemistry (Mosc.)*, **17**, 4277–4285.

Lewis,B.A. *et al.* (2011) PRIDB: a Protein-RNA interface database. *Nucleic Acids Res.*, **39**, D277–282.

Lunde,B.M. *et al.* (2007) RNA-binding proteins: modular design for efficient function. *Nat. Rev. Mol. Cell Biol.*, **8**, 479–490.

Maris,C. *et al.* (2005) The RNA recognition motif, a plastic RNA-binding platform to regulate post-transcriptional gene expression. *FEBS J.*, **272**, 2118ñ2131.

Marsh,J.A. (2013) Buried and accessible surface area control intrinsic protein flexibility. *J. Mol. Biol.*, **425**, 3250–3263.

Nagano,K. (1973) Logical analysis of the mechanism of protein folding. I. Predictions of helices, loops and beta-structures from primary structure. *J. Mol. Biol.*, **75**, 401–420.

Nagarajan,R. and Gromiha,M.M. (2014) Prediction of RNA Binding Residues: An Extensive Analysis Based on Structure and Function to Select the Best Predictor. *PLoS ONE*, **9**, e91140.

Nakashima,H. *et al.* (1990) Distinct character in hydrophobicity of amino acid compositions of mitochondrial proteins. *Proteins*, **8**, 173–178.

Palau,J. *et al.* (1982) Protein secondary structure. Studies on the limits of prediction accuracy. *Int. J. Pept. Protein Res.*, **19**, 394–401.

Pawar,A.P. *et al.* (2005) Prediction of 'aggregation-prone' and 'aggregation-susceptible' regions in proteins associated with neurodegenerative diseases. *J. Mol. Biol.*, **350**, 379–392.

Pedregosa,F. *et al.* (2011) Scikit-learn: Machine Learning in Python. *J. Mach. Learn. Res.*, **12**, 2825ñ2830.

Radzicka,A. *et al.* (1988) Influences of solvent water on protein folding: free energies of solvation of cis and trans

peptides are nearly identical. *Biochemistry (Mosc.)*, **27**, 4538–4541.
R Development Core Team (2008) R: A Language and Environment for Statistical Computing R Foundation for Statistical Computing, Vienna, Austria.
Rossum,G. (1995) Python Reference Manual CWI (Centre for Mathematics and Computer Science), Amsterdam, The Netherlands, The Netherlands.
Sha,B. and Luo,M. (1997) Structure of a bifunctional membrane-RNA binding protein, influenza virus matrix protein M1. *Nat. Struct. Mol. Biol.*, **4**, 239–244.
Sweet,R.M. and Eisenberg,D. (1983) Correlation of sequence hydrophobicities measures similarity in three-dimensional protein structure. *J. Mol. Biol.*, **171**, 479–488.
Terribilini,M. *et al.* (2006) Prediction of RNA binding sites in proteins from amino acid sequence. *RNA N. Y. N*, **12**, 1450–1462.
Walia,R.R. *et al.* (2014) RNABindRPlus: a predictor that combines machine learning and sequence homology-based methods to improve the reliability of predicted RNA-binding residues in proteins. *PloS One*, **9**, e97725.
Wang,L. *et al.* (2010) BindN+ for accurate prediction of DNA and RNA-binding residues from protein sequence features. *BMC Syst. Biol.*, 4 Suppl 1, S3.
